# What Goes Wrong during Early Development of Artificially Reproduced European Eel *Anguilla anguilla*? Clues from the Larval Transcriptome and Gene Expression Patterns

**DOI:** 10.3390/ani11061710

**Published:** 2021-06-08

**Authors:** Pauline Jéhannet, Arjan P. Palstra, Leon T. N. Heinsbroek, Leo Kruijt, Ron P. Dirks, William Swinkels, Hans Komen

**Affiliations:** 1 Animal Breeding and Genomics, Wageningen University & Research, 6708 PB Wageningen, The Netherlands; pauline.jehannet@wur.nl (P.J.); leo.kruijt@wur.nl (L.K.); hans.komen@wur.nl (H.K.); 2 Wageningen Eel Reproduction Experts B.V., 3708 AB Wageningen, The Netherlands; leon.heinsbroek@wur.nl; 3 Future Genomics Technologies B.V., 2333 BE Leiden, The Netherlands; dirks@futuregenomics.tech; 4 DUPAN Foundation, 6708 WH Wageningen, The Netherlands; wswinkels@dupan.nl

**Keywords:** European eel *Anguilla anguilla*, aquaculture, immune system, osmoregulation, morphogenesis, RNA-Seq

## Abstract

**Simple Summary:**

Closing the life cycle of the European eel in captivity is urgently needed to gain perspective for the commercial production of juvenile glass eels. Larvae are produced weekly at our facilities, but large variations in larval mortality are observed during the first week after hatching. Although much effort has been devoted to investigating ways to prevent early larval mortality, it remains unclear what the causes are. The aim of this study was to perform a transcriptomic study on European eel larvae in order to identify genes and physiological pathways that are differentially regulated in the comparison of larvae from batches that did not survive for longer than three days vs. larvae from batches that survived for at least a week up to 22 days after hatching (non-viable vs. viable larvae). In contrast to earlier published studies on European eel, we conclude that larvae exhibit immune competency. Non-viable larvae initiated an inflammatory and host protection immune response and tried to maintain osmoregulatory homeostasis. As a perspective, microbial control and salinity reduction might benefit eel larvae in terms of lower mortality and improved development by lowering the costs of immune functioning and osmoregulation.

**Abstract:**

In eels, large variations in larval mortality exist, which would impede the viable production of juvenile glass eels in captivity. The transcriptome of European eel larvae was investigated to identify physiological pathways and genes that show differential regulation between non-viable vs. viable larvae. Expression of genes involved in inflammation and host protection was higher, suggesting that non-viable larvae suffered from microbial infection. Expression of genes involved in osmoregulation was also higher, implying that non-viable larvae tried to maintain homeostasis by strong osmoregulatory adaptation. Expression of genes involved in myogenesis, neural, and sensory development was reduced in the non-viable larvae. Expression of the major histocompatibility complex class-I (*mhc1*) gene, M-protein (*myom2*), the dopamine 2B receptor (*d2br*), the melatonin receptor (*mtr1*), and heat-shock protein beta-1 (*hspb1*) showed strong differential regulation and was therefore studied in 1, 8, and 15 days post-hatch (dph) larvae by RT-PCR to comprehend the roles of these genes during ontogeny. Expression patterning of these genes indicated the start of active swimming (8 dph) and feed searching behavior (15 dph) and confirmed immunocompetence immediately after hatching. This study revealed useful insights for improving larval survival by microbial control and salinity reduction.

## 1. Introduction

European eel cannot be propagated. Eel farms depend on wild-caught glass eels that are grown to marketable size. Closing the production cycle of this species is urgently needed to ensure the supply of young juvenile glass eels. European male eels were first matured by injection of urine from pregnant women ([1]; containing human chorionic gonadotropin–hCG), females by hypophysation (i.e., weekly injection of pituitary extracts) in the 1960s [2], and eggs were first fertilized in 1980 [3], after which the first larvae were produced in the early 1980s [4]. Although several groups can now produce larvae of European eel on a regular basis [5,6,7,8], massive mortality often occurs [9,10], particularly during the first week after hatching. Survival rates during the first week vary widely from 0 to 90% in European eels [10]. The life cycle for the Japanese eel has been closed [11], but still, first week survival ranges from 15 to 92% [12]

For most marine fish species in aquaculture, the high and unpredictable mortality in larvae remains a challenging problem that needs to be solved [13]. Although egg quality and larval nutrition have been considered as the main causes of larval mortality, these factors cannot explain the considerable variation in mortality between full sibling groups that are treated equally [13,14]. Accumulating evidence suggests that detrimental fish–microbe interaction is the main cause of larval mortality in marine species like turbot, halibut, plaice, and sea bass [14]. In teleost fish, early larvae mostly rely on a complex network of innate defense mechanisms (physical barriers, cellular defenses, and inflammatory cytokines) to orchestrate a rapid immune response against the hostile environment (reviewed by [13]). For European eels, it has been recently hypothesized that early larvae are immunocompromised and highly sensitive to pathogens [15]. Besides defense against antigens, early larvae need to cope with seawater salinities and thus face ion invasion and dehydration. In teleost fish, early larvae are already able to osmoregulate at hatching and this ability increases with age, as reviewed by [16]. Early larvae of the Japanese eel *A. japonica* possess numerous ionocytes with multicellular complexes that are essential for salt secretion [17] and they drink as early as hatching to compensate for water loss [18].

Besides coping with the external environment, early fish larvae need to grow, develop, and survive. During the first 12 to 20 dph, depending on the temperature, European eel larvae feed on yolk reserves (depleted around 14 dph at 20 °C; [6]). Quantity and quality of the yolk and oil droplet might affect larvae survival in marine fish [19]. In European eels, the rate of yolk consumption was the same between larval batches, but larvae with more yolk reserves had a survival advantage over those having smaller ones [6]. In teleost fish, yolk resorption coincides with the development of the digestive system indicating that most yolk is used for organogenesis [6,20,21,22]. In teleost fish, the predominant changes in gene expression during early larval development are related to neural development, sensory system, muscular development, ossification, digestive function and the regulation of metabolic pathways reviewed by [23].

In European eels, neural development starts as early as embryogenesis since brain rudiments are already observed at 22 h post fertilization at 20 °C [6]. Although eye rudiments are observed in embryos, the visual system becomes functional until eye pigmentation at 8 dph in European eels at 20 °C [6] and in Japanese eels at 19 °C [24]. Therefore, the most prominent sensory system in new hatchlings might be that of mechanoreception since New-Zealand eel larvae respond to movements of the beakers that they are in [25]. Like most fish species reviewed by [26], the digestive system of eel larvae is still largely underdeveloped at hatching [6,27]. In new hatchlings, the digestive tract is close to the oil droplet and develops into a straight and narrow tube at 6 dph at 20 °C [28]. The mouth is observed at 3 dph, undergoes profound changes at 5 dph, develops lower and upper jaws at 8 dph [6] and starts moving when the musculoskeletal anatomy has sufficiently developed at 12 dph at 20 °C [29]. A recent study in Japanese eel showed that dentary and maxillary ossification at the jaw starts at 10 dph between 23–26 °C [30]. Expression of appetite (*ghrelin* and *cholecystokinin*) and digestion (*amylase*, *trypsin* and *lipase*) enzymes was all detected at hatching and increased through endogenous feeding to reach increased values prior to or at the onset of exogeneous feeding at 14 dph in European eels at 18–20 °C [28,31,32]. In fish larvae, but also in other vertebrates, the metabolic rate influences the amount of energy available and therefore larvae survival. Eel larvae have a unique body composed mainly of glycosaminoglycans that are non-metabolizing compounds [33]. In sharp contrast with other species, eel larvae can grow to large size with minimal metabolic activity [33,34].

Although much effort has been devoted to understanding larval mortality (European eels: [6,15,35,36]; Japanese eels: [37,38,39,40,41]), it remains unclear what goes wrong during early ontogeny of artificially reproduced eels. With recent advances in sequencing technology, transcriptomic approaches have been widely used to understand marine fish larvae development [23]. In Japanese eels, deep RNA sequencing has been recently applied for studying processes of digestion and absorption in early life stages [42] and maternal transcripts in good and poor quality eggs [43]. In European eels, there is still a lack of transcriptomic data covering the early ontogeny of European eels and filling this gap would be essential to identify pathways and genes marking important critical events during early ontogeny.

The aim of this study was to perform a transcriptomic study on European eel larvae to identify genes and physiological pathways that show differential regulation in non-viable vs. viable larvae. Larvae collected at 1 dph from batches that survived for at least a week were classified as viable larvae, while those from batches that survived less than 3 dph were classified as non-viable larvae. From the RNA-seq data, differentially expressed genes (DEGs) were analyzed between non-viable vs. viable larvae to understand what goes wrong during early ontogeny in the first week following hatching. Furthermore, we investigated the expression patterns of several highly differentially expressed genes (*mhc1, myom2*, *d2br*, *mt1r* and *hspb1*) by RT-PCR in 1, 8, 15 dph larvae to further comprehend the changes in molecular regulation of processes they are involved in.

## 2. Materials and Methods

### 2.1. Broodstock

Female broodstock eels were transferred as elvers from Palingkwekerij Koolen BV (Bergeijk, The Netherlands) to the animal experimental facilities of Wageningen University & Research (CARUS, Wageningen, The Netherlands). Elvers were feminized by feeding them with 17β-estradiol (E2) coated pellets over a 6 month-period [44]. After an additional 6 months of feeding them with a custom-made broodstock diet, eels of ~400 g were selected, transferred to seawater (Tropic Marine, 36 ppt) and fed no longer. For 2 months, eels were then subjected to simulated migration: constant swimming in the dark at daily alternating temperatures between 10 and 15 °C to make them silver [45]. Also wild silver females (250–800 g) and males (100–200 g; Van Harinxma Canal, The Netherlands) were used as broodstock.

### 2.2. Induction of Gametogenesis

For induction of gametogenesis, females were transferred to 373 L-tanks (16 °C, 36 ppt) and treated with a steroid implant for an additional 2 months [46,47]. Females were then weekly injected with carp pituitary extracts (CPE) at a dose of 20 mg·kg^−1^ over a period of 7–15 weeks to induce vitellogenesis and oocyte maturation, and injected with 17*α*,20*β* -dihydroxy-4-pregnen-3-one (DHP) at a dose of 2 mg·kg^−1^ to induce ovulation following previously described procedures [48,49]. Females were then placed in a tank in which the temperature was gradually increased from 18 to 20 °C and when females were ready to spawn after 11–15 h after DHP injection, eggs were stripped by applying gentle pressure along the abdomen.

Male eels were matured by a single hCG injection [50]. Twenty-four hours before use, males were checked for spermiation by applying gentle pressure along the abdomen. Spermiating males (*n* = 3–6) received another hCG injection to enhance high quality sperm production [51]. Before stripping the eggs, sperm was collected by stripping these males and used for fertilization.

### 2.3. Fertilization and Egg Rearing

Eggs were collected in dry bowls and gametes were gently mixed. Artificial seawater (Tropic Marine, 36 ppt, 18 °C) prepared by using reverse osmosis filtration was added to the bowls for gamete activation and fertilization for 5 min. Eggs were then incubated under dark conditions in 3L-beakers (*n* = ~1000 eggs per liter) filled with the previously described artificial seawater. Every 12 h, dead material was removed and half volume of the water was refreshed. After hatching (~60 hpf), larvae were stocked in plankton nets hanging in conic tanks connected to a 338 L recirculating system with artificial seawater (36 ppt, 18 °C) at an exchange rate of 5%/d. Larval longevity (i.e., the number of dph that larvae survived) was monitored for each batch.

### 2.4. Larvae Collection

**Figure 1:**
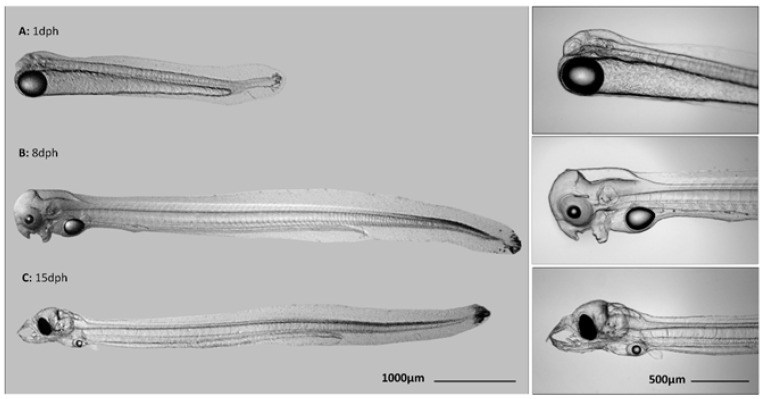
Larvae of European eel *Anguilla anguilla* at (**A**) one day post hatch (dph); (**B**) 8 dph, and (**C**) 15 dph. At 1 dph, new hatchlings hang in the water column with large yolk-reserves. At 8 dph, larvae start swimming and develop upper and lower jaws. Eyes become pigmented. At 15 dph, larvae swim actively. Yolk-reserves are almost depleted, the protruding teeth are formed, and larvae should start exogenous feeding. Eyes are completely pigmented.

Larvae (*n* = 10) were randomly collected and pipetted in RNAlater (ThermoFisher, Waltham, MA, USA) at 1 dph for later RNA-Seq analysis. Larvae that survived less than 3 dph were classified as coming from a non-viable batch, while those that survived for at least a week were classified as viable. Larvae that were used for RNA-Seq analysis are listed in Table 1. For gene expression analysis, larvae (*n* = 10) were collected at 1 dph, 8 dph, and 15 dph (Figure 1) and pipetted in RNAlater. In general, 1 dph larvae did not show malformations in our study, which is in sharp contrast with the 8 and 15 dph larvae. Therefore, only larvae that did not show aberrant malformations (e.g., broken jaw, curved tail) were selected at 8 dph and 15 dph.

### 2.5. RNA-Sequencing

RNA-Seq was performed on the RNA of non-viable larvae (*n* = 3 samples) and viable larvae (*n* = 3 samples). RNA from larvae was isolated using a miRNeasy Kit (Qiagen). RNA concentrations measured with the Bio-Analyzer ranged between 38.7 and 137 ng μL^−1^ and RIN values were generally 7.5 to 9.4. All RNA-Seq libraries were sequenced on an Illumina NovaSeq6000 sequencer as Illumina Paired-end 2 × 150 nt run (10 Mreads; 3 Gb), according to the manufacturer’s protocol. Illumina multiplexed RNA-Seq libraries were prepared from 0.5 μg total RNA using the Illumina TruSeq Stranded mRNA Library Prep according to the manufacturer’s instructions (Illumina Inc., San Diego, CA, USA). Image analysis and base calling were done by the Illumina pipeline. A total of 16 up to 32 million raw read counts were derived per sample. Quantitative analysis of the RNA-Seq datasets was performed by alignment of reads against the European eel *Anguilla anguilla* reference genome (https://www.ncbi.nlm.nih.gov/genome/10841?genome_assembly_id=59496 accessed on 20 May 2014) using TopHat (version 2.0.13; [52] Center for Computational Biology at Johns Hopkins University, Baltimore, MD, USA; options: tophat -o “file_address” -i 50 -p 10 --library-type fr-unstranded --b2-very-sensitive --no-coverage-search --GTF Ref_genome.gff Ref_genome R1.fastq R2.fastq) and 9.8–16.7 million (53–62%) of the RNA-Seq reads could be mapped. Reference alignment was done, and the resulting files were filtered using SAMtools (Wellcome Genome Campus, Hinxton, Cambridgeshire, UK; version 1.2 using htslib 1.2.1; [53], secondary alignments were removed using the command: samtools view -h -o file.sam -F 0x0100 file.bam) to exclude secondary alignment of reads (~5.3%). For statistical comparison of gene expression levels between groups, aligned fragments per predicted gene were counted from SAM alignment files using the Python package HTSeq (https://readthedocs.org/projects/htseq/; version 0.6.1p1) [54]. In order to make comparisons across samples possible, these fragment counts were corrected for the total amount of sequencing performed for each sample. As a correction scaling factor, we employed library size estimates determined using the R/Bioconductor (https://bioconductor.riken.jp/packages/3.4/bioc/html/DESeq.html; release 3.3.2) package DESeq [55]. Read counts were normalized by dividing the raw counts obtained from HTSeq by its scale factor. Aligned reads were processed using DESeq whereby treatment groups were each compared with the control group. Raw RNA-Seq data (reads) have been submitted to NCBI’s SRA database with reference PRJNA735388 (http://www.ncbi.nlm.nih.gov/bioproject/735388; SAMN19580333-SAMN19580338; Temporary Submission ID: SUB9805749; Release date: 6 June 2021). The comparison non-viable vs. viable larvae at 1 dph was analyzed to assess differential gene expression and their functional clustering during early ontogeny by GO analysis using UniProt (https://www.uniprot.org/).

### 2.6. Gene Expression

#### 2.6.1. Gene Description and Primer Designs

From the RNA-Seq data, differentially expressed genes marking important functional processes were selected and further examined by RT-PCR in the 1, 8, and 15 dph larvae. These genes were the major histocompatibility complex class I (*mhc1*), M-protein (*myom2*), the dopamine 2B receptor (*d2br*), the melatonin receptor (*mtr1*), and heat-shock protein beta-1 (*hspb1*). Primers were designed on the basis of the cDNA contig sequences of the Illumina assembly of European eel. Primers previously developed for *d2br* [56] were aligned with the cDNA contigs to check whether the primers shared 100% sequence identity between the cDNA contigs and oligonucleotide sequence. Primers previously developed for the housekeeping gene *60s ribosomal protein l36 (l36)* was used [56,57]. Primers used for qPCR analysis and designed using Primer3 v.0.4.0 [58,59] are listed in Table 2.

#### 2.6.2. RNA Isolation

Total RNA was isolated from larvae (*n* = 10) collected at 1, 8, and 15 dph larvae with Trizol Reagent as described by the manufacturer (Invitrogen, California, USA). RNA concentration measured with the nanodrop was 1333 ± 832, 407 ± 2225, and 185 ± 80 ng μL^−1^ at 1, 8, and 15 dph, respectively. Possible traces of DNA were digested with the ISOLATE II RNA Mini Kit (Bioline, London, UK). Complementary DNA (125 ng μL^−1^) was generated from RNA using dNTPs and random primers with Superscript III (ThermoFisher, Waltham, MA, USA). RNA purity was assessed by spectrophotometry; the 260:280 ratios were 2.1 ± 0.1 and the 260:230 ratios were 1.5 ± 0.4. RNA integrity was checked on an Agilent bioanalyzer 2100 (Agilent technologies, CA, USA) and no RNA breakdown was observed on the gel.

#### 2.6.3. Quantitative RT-PCR

Quantitative real-time PCR was performed with SensiFAST™ SYBR^®^ Lo-ROX Ki (Bioline, London, UK) on a QuantStudio^TM^-5 Real-Time PCR system (ThermoFisher, Waltham, MA, USA). Reactions were heated for 2 min at 95 °C followed by 40 cycles of denaturation at 95 °C for 5 s and annealing temperature at 60–64 °C for 20 s. Melting curves from 60 °C to 95 °C holding during 20 s and 1 s, respectively, were generated to check for primer-dimer artifacts and reaction specificity. Primer efficiencies were determined by generating standards for the housekeeping gene *60s ribosomal protein l36 (l36)* and selected target genes (*d2br*, *mtr1*, *hspb1*, *mhc1*, *myom2*). Standard curves were generated by diluting cDNA at 1:5 for *l36* (Ct_5_^−1^: 21.9; Ct_5_^−2^: 24.2; Ct_5_^−3^: 26.6; Ct_5_^−4^: 29.1; Ct_5_^−5^: 31.8), *mhc1* (Ct_5_^−1^: 22.9; Ct_5_^−2^: 25.3; Ct_5_^−3^: 27.7; Ct_5_^−4^: 30.2; Ct_5_^−5^: 32.9), *myom2* (Ct_5_^−1^: 25.8; Ct_5_^−2^: 27.9; Ct_5_^−3^: 30.2; Ct_5_^−4^: 32.8; Ct_5_^−5^: 35.2; Ct_5_^−6^: 36.6) and at 1:2 for *mtr1* (Ct_2_^−1^: 28.8; Ct_2_^−2^: 30.1; Ct_2_^−3^: 31.2; Ct_2_^−4^: 32.0; Ct_2_^−5^: 33.2), *d2br* (Ct_2_^−1^: 28.5; Ct_2_^−2^: 29.3; Ct_2_^−3^: 30.3; Ct_2_^−4^: 31.7; Ct_2_^−5^: 32.1; Ct_2_^−6^: 33.7; Ct_2_^−7^: 34.9) and *hspb1* (Ct_2_^−1^: 25.9; Ct_2_^−2^: 26.8; Ct_2_^−3^: 27.9; Ct_2_^−4^: 28.7; Ct_2_^−5^: 29.6; Ct_2_^−6^: 31.4). R^2^ values and efficiency for all standard curves were >0.98 and 90–110%, respectively (c.f., MIQE guidelines in [62]). Data were expressed as fold change by using the 2_T_−^ΔΔC^ method [63]. Transcript levels of each target gene were normalized over *l36* since expression levels were not significantly different between groups (*p* > 0.96).

#### 2.6.4. Statistical Analysis

Means of normalized copy numbers of each target gene were compared between 1, 8, and 15 dph larvae using the Kruskal–Wallis test followed by a pairwise Wilcoxon-test for multiple comparisons among groups. Data are expressed as mean ± standard deviation and differences were considered significant at *p* < 0.05. Statistical analysis was performed in R (version 3.2.4; R foundation for statistical computing, Vienna, Austria).

## 3. Results

### 3.1. Eel Larvae Transcriptomics

The comparison of non-viable vs. viable larvae yielded 36,160 transcripts that were associated with NCBI *A. anguilla* genes. The comparison non-viable vs. viable showed that 35,802 transcripts were not differentially expressed (Table S1). Of these transcripts, several genes were highly abundant (based on the mean number of reads). Many genes involved in innate immunity (e.g., *toll-like receptor 7*, *Toll-interacting protein, complement C3*) were highly abundant in 1 dph larvae. In addition, several transcripts associated with osmoregulation (e.g., *claudin-23*, *claudin-5, claudin-1, claudin-7*), muscular development (e.g., *myosin heavy chain*, *troponin C*, *troponin T*, *collagen alpha*), neural development (e.g., *neurabin-1-like*, *neurabin-2*), sensory development (e.g., *melanopsin-A-like*, *beta-crystallin B3*, *ketimine reductase mu-crystallin*), and in Wnt signaling (e.g., *Wnt-bd domain containing protein*, *WNT1-inducible-signaling pathway protein 1-like*, *protein Wnt-11*) were highly abundant. Additionally, several genes encoding digestive enzymes relating to lipid hydrolysis showed high abundancy (e.g., *lipoprotein lipase*, *monoglyceride lipase*, *endothelial lipase, group XIIA secretory phospholipase A2*, *cytosolic phospholipase A2*) in 1 dph larvae. One transcript related to protein hydrolysis (*cationic trypsin-like)* was highly abundant in 1 dph larvae, while several transcripts related to carbohydrate hydrolysis were moderately present (e.g., *pancreatic alpha amylase-like*, *alpha amylase*). Furthermore, several transcripts associated with hyaluronan metabolism (*inter-alpha-trypsin inhibitor heavy chain H3-like*, *inter-alpha-trypsin inhibitor heavy chain H2*, *inter-alpha-trypsin inhibitor heavy chain H5-like*) were highly abundant in 1 dph larvae.

The comparison non-viable vs. viable larvae showed significant differential expression of 358 genes at *p* < 0.05 (Table S2). Of these 358 differentially expressed genes (DEGs), expression of 123 genes showed high fold change expression (e.g., upregulated expression) and expression of 235 genes had low fold change (e.g., down-regulated expression). Among these DEGs were several genes involved in the immune response (Table S3) and associated with GO terms such as pathogen recognition-destruction (*mhc1, complement component C7*, *pentraxin*), inflammation (*interleukin 17-C*, *nlrp12*) and host protection (*complement factor H*, *arginase-2*, *leukocyte elastase inhibitor*, *complement decay-accelerating factor*) on the biological process level. From these DEGs, seven out of nine showed high fold change expression in non-viable vs. viable larvae, as shown in Table 3. Additionally represented were several DEGs involved in osmoregulation (Table 4). From these DEGs, five out of six showed high fold change expression in non-viable vs. viable larvae. These DEGs were associated with osmosensing and Ca^2+^ homeostasis (*extracellular calcium-sensing receptor-like*), gill tissue reshaping (*claudin-4*), hyperosmolarity compensation (*sodium/myo-inositol cotransporter-like*), water/salt absorption (*guanylin precursor*), and salt secretion (*claudin-10*). Furthermore, additional important DEGs were involved in morphogenesis (Table 5) and associated with GO terms such as muscle development (*hspb1*, *cGMP*, *troponin I*, *myom2*), neural development (*Pro-neuregulin-1*, *CUB and sushi domain-containing protein 3*, *homeobox protein Lhx2*, *homeobox protein otx2, homeobox protein pnx*, *disintegrin and metalloproteinase domain-containing protein 22*, *mt1r*, *Protocadherin-16*, *d2br*), sensory system (*vertebrate ancient opsin-like*, *LIM/homeobox protein Lhx2*, *norrin*, *putative transmembrane channel-like protein 1*), Wnt signaling (norrin-like, receptor-type tyrosine-protein phosphatase O, CXXC-type zinc finger protein 4), and in various aspects of morphogenesis (*T-box transcription factor TBX1*, *homeobox protein DLX-6*, *homeobox protein DLX-2*).

### 3.2. Eel Larvae Temporal Expression

**Figure 2:**
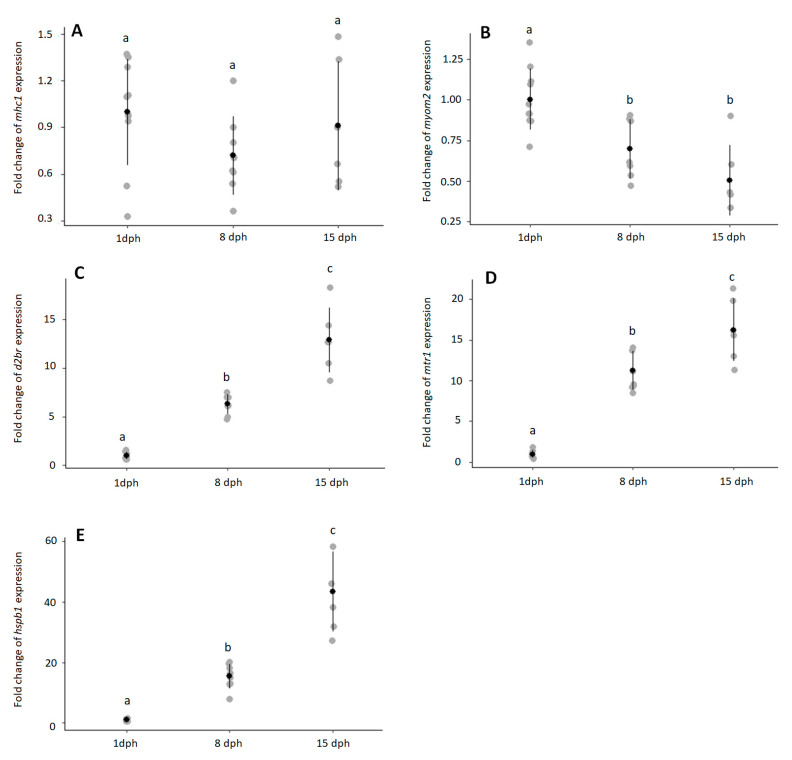
Temporal expression in European eel larvae at 1 (*n* = 10), 8 (*n* = 8) and 15 (*n* = 6) dph of (**A**) major-histocompability complex class I *mhc1* (*p* > 0.2295); (**B**) M-protein *myom2* (*p* < 0.0019); (**C**) dopamine 2B receptor *d2br* (*p* < 6.12 × 10^−5^); (**D**) melatonin receptor *mtr1* (*p* < 8.75 × 10^−5^); (**E**) heat-shock protein beta-1 *hspb1* (*p* < 6.12 × 10^−5^). Outliers (>2 Standard Deviation—SD) were detected at 1 dph for *d2br* and *hspb1* and at 8 dph for *myom2*, which were removed before running the statistical analysis. Expression is shown as fold change vs. 1 dph larvae. Individual values are given in grey, averages with standard deviation in black. Different letters (a, b, c) indicate statistical differences between groups (Kruskal–Wallis followed by a pairwise Wilcoxon-test, *p* < 0.05).

Expression of *mhc-I* was high throughout larval development with low Ct values in 1, 8, and 15 dph larvae (1 dph: 24.2 ± 0.65; 8 dph: 24.6 ± 0.98; 15 dph: 24.0 ± 0.66). Expression of *mhc-I* did not change with larval development (Figure 2A, *p* > 0.2295). In contrast, *myom2* expression decreased during early ontogeny (Figure 2B, *p* < 0.0019) and was downregulated in 8 dph (*p* < 0.0102) and 15 dph (*p* < 0.0052) larvae. Transcript levels of *d2br* increased during early ontogeny (Figure 2C, *p* < 6.12 × 10^−5^). Expression of *d2br* was approximately 6-fold and 13-fold higher in 8 dph and 15 dph, respectively, when compared with 1 dph larvae. Like *d2br*, *mrt-1* expression increased with larval development (Figure 2D, *p* < 8.74 × 10^−5^). Expression of *mrt-1* was approximately 11-fold and 13-fold higher in 8 dph and 15 dph, respectively, when compared with 1 dph larvae. Transcript levels of *hspb1* significantly increased during early ontogeny (Figure 2E, *p* < 6.12 × 10^−5^) and fold change peaked in 15 dph larvae with over 43-fold.

## 4. Discussion

In European and Japanese eels, but also in other marine fish species such as Bluefin Tuna *Thunnus orientalis* [64], an important bottleneck is the stable production of viable larvae. In aquaculture fish, larval quality is influenced by many factors such as broodstock nutrition, system conditions, and spawning induction [65]. Although much attention has been paid to optimizing the rearing conditions in eel larviculture [37,38,39,40,41,42,43], rapid decrease in larvae survival rates around 2–5 dph is often observed. In this transcriptomic study, clues about larval mortality during the first week after hatching were obtained by comparing non-viable vs. viable larvae at 1 dph. In addition, the temporal expression of highly differentially expressed genes that mark the innate and adaptive immune response (*mhc-I*), muscle growth (*myom2),* movement (*d2br*, *mtr1*), and stress (*hspb1*) was investigated in 1, 8, and 15 dph larvae to better comprehend their role during early ontogeny of the European eel.

### 4.1. Immune Response

In our study, numerous transcripts associated with the immune response were highly abundant but not differentially expressed in non-viable vs. viable larvae at 1 dph. Transcripts associated with innate immunity were more abundant than those involved in adaptive immunity. These findings are consistent with another recent study on European eel [15] and the ontogeny of larval immunity in other teleost fish species reviewed by [13]. GO analysis of DEGs showed that immune-related terms were abundant in non-viable vs. viable larvae. Most of these genes had increased expression following immune challenge experiments in fish (*mhc1:* [66]; *C7*: [67,68,69]; *complement factor H:* [70,71]; *arginase-2:* [72]; *leukocyte elastase inhibitor:* [73]; *nrlp12:* [74]; *complement decay-accelerating factor:* [75]; *interleukin-17C:* [76,77,78]), suggesting an important role in the immune response. Bacterial infections are recognized as one of the most frequent causes affecting larvae survival in fish [13,14]. In eel larviculture, the use of antibiotics and disinfection treatments has been shown to increase larvae survival in eels [9].

In our study, two genes related to pathogen recognition and destruction showed very low (negative) fold changes at 1 dph in non-viable vs. viable larvae. Among them, *mhc1* showed the lowest fold change expression in non-viable larvae (−18-fold). Mhc-I is essential for presenting peptides from intracellular pathogens to cytotoxic CD8+ T-cells in innate and adaptive immunity [79]. *Complement component C7* also showed low fold change expression in non-viable vs. viable larvae (−8-fold). C7 is an essential member of the membrane attack complex that forms transmembrane channels to induce pathogen cytolysis [80]. *Pentraxin* showed a slightly higher expression in non-viable vs. viable larvae (3-fold). Pentraxin is a classic pattern recognition molecule used to defend against bacterial infection in innate immunity in tongue sole [81]. The low fold changes of genes related to pathogen recognition-destruction suggest that eel larvae exhibit immune competency, which is reduced in non-viable larvae at 1 dph.

Two genes in our study that were related to inflammation showed high fold changes in non-viable vs. viable larvae at 1 dph. Expression of *interleukin-17C*, which showed the highest fold change (29-fold), is essential for regulating the inflammatory response and host defense via the NF-kB pathway in large yellow croaker [78]. Nlrp12 regulates the inflammatory response by operating within inflammasomes [82]. Although inflammatory responses are essential for protecting early larvae from pathogens [13], excessive inflammation can cause severe damage. Therefore, inflammation needs to be finely tuned to maintain a balance between host protection and inflammatory diseases. Several genes related to host protection showed high fold change expression in non-viable vs. viable larvae. Non-viable larvae may attempt to limit pathogen invasion as indicated by: (i) genes that code for regulatory proteins that protect self-cells from autologous attacks such as *complement factor-H* and *complement decay-accelerating factor* [83]; (ii) *leukocyte elastase inhibitor* that limits host damage during inflammation, apoptosis, and pathogen destruction [84]; and (iii) *arginase-2* that is associated with the presence of ‘healing’ macrophages in carp [72,85]. In conclusion, the high fold change expression of genes related to inflammation and host protection suggests that non-viable larvae had initiated immune responses toward invading pathogens.

When considering that numerous transcripts associated with the immune response (e.g., complement component, toll receptors) were highly abundant at 1 dph but not differentially expressed between non-viable vs. viable larvae in our RNA-Seq data, we can conclude that the (innate) immune system plays an important role in early larval development, already just after hatching. In our study, *mhc1* was highly expressed in 1, 8, and 15 dph larvae, but did not change its expression through larval development. In accordance with our results, *mhc1* showed an early and high expression in rainbow trout larvae [86] and was already detected at 1 dph in common carps [87]. The high abundance of many genes related to the immune response and the high expression of *mhc1* during early ontogeny of eel larvae in our study provides supporting evidence against the hypothesized immunocompromised eel larvae of Miest et al. [15]. These authors suggested that eel larvae were immunocompromised since the expression of key genes involved in the immune system showed low expression between hatching (0 dph) and teeth formation (8 dph). In our study, eel larvae exhibited immune competency but non-viable larvae seem to be more sensitive to microbial infections. As suggested by Sørensen et al. [9], microbial controls through disinfection treatments in combination with microbial management would be essential to improve larvae survival in eels.

### 4.2. Osmoregulation

In our study, numerous transcripts associated with osmoregulation (e.g., claudins) were highly abundant, but not differentially expressed in non-viable vs. viable larvae at 1 dph. Previous studies have shown that eel larvae are able to osmoregulate just after hatching (for *A. japonica* [18] and also for other fish species reviewed by [16]). GO analysis of DEGs showed that osmoregulation-related terms were abundant in non-viable vs. viable larvae, which suggests a difference in maintaining ionic and osmotic balance in 100% SW. Lee et al. [88] showed that the tissue osmolality of Japanese eel larvae (360 to 540 mOsm/kg·H_2_O) was actively regulated to stay at lower osmolality than seawater osmolality (about 1000 mOsm/kg·H_2_O). Early larvae osmoregulate by ingesting water as early as hatching to prevent osmotic water loss [18] and possess chloride cells on their yolk-sac membrane and integument to maintain their ionic balance [17]. It has been shown that reducing salinity enhanced larval survival in anguilloid species (Japanese eel in [89]; European eels in [90]). Even deformed larvae were able to survive in 50% SW [89]. These findings show that eel larvae in seawater invest much of their available energy on osmoregulation, which would become available for other vital processes when lowering the salinity. Although salinity reduction improves larvae survival, an increased number of larvae with pericardial oedema and notochord deformities have been observed under these circumstances, both in European and Japanese eels [89,90,91].

Expression of the *extracellular calcium-sensing receptor*, which showed the greatest difference in non-viable vs. viable larvae in our RNA-Seq data (39-fold), has been suggested to be essential for calcium homeostasis and osmosensing in fish [92]. The high fold change of this gene in non-viable vs. viable larvae suggests that non-viable larvae suffer from membrane damage and leakage and try to compensate the permeability by strong osmoregulatory adaptations, which is also indicated by the expression of several other genes: (i) *guanylin precursor* that codes for a prohormone that is cleaved within the intestinal lumen or kidney tubules into small peptides that regulate water and salt absorption in seawater (SW) in eels [93,94,95]; (ii) *claudin-10 isoforms* that code for proteins that are associated with salt secretion in SW in euryhaline species [96]; and (iii) *sodium/myo-inositol cotransporter* that codes for a protein that allows for the accumulation of osmolytes within cell types to compensate for hyperosmolarity in mammalian systems [97]. *Claudin-4*, another osmoregulatory gene, had high fold change expression in non-viable vs. viable larvae. Upregulated *claudin-4* expression was associated with freshwater acclimation in southern flounder *Paralichthys lethostigma* [98]. The high fold-change of *claudin-4* might reflect a dysfunction of the non-viable larvae to osmoregulate in SW since an increase of claudin-4 is essential for the formation of deeper tight junctions to reduce ion permeability; a crucial facet of freshwater osmoregulation [98]. Therefore, important osmoregulatory genes are differentially expressed in non-viable vs. viable larvae, but it is worth noticing that differential expression of these genes might be a symptom, rather than a cause, of dying.

### 4.3. Myogenesis, Neurogenesis, and Sensory Development

As could be expected for early larvae, numerous transcripts associated with morphogenesis were highly abundant in both non-viable and viable larvae at 1 dph. Genes related to myogenesis were highly abundant (based on the mean copy number) in both non-viable and viable larvae at 1 dph. High abundancy of genes related to myogenesis in non-viable larvae might be related to stratified hyperplasia that allows for the increase in the number of muscle fibers during early ontogeny [23]. Only three genes related to muscle development (*cGMP, troponin-I, myom2*) showed very low negative fold changes at 1 dph in non-viable vs. viable larvae. While Myom2 is essential for the sarcomeric organization of vertebrate striated muscle [99], Troponin-I, and cGMP are involved in muscle contraction [100,101]. The low fold changes of these genes in non-viable vs. viable larvae suggests that muscle functionality might be affected in non-viable larvae. The temporal expression of *myom2* was studied in 1, 8, and 15 dph larvae and was found to decrease during early ontogeny. Myom2, or M-protein, is expressed in cardiac and skeletal muscle but its exact function in fish larvae is not known. We assume that *myom2* is related to muscle growth and development since its expression decreased toward 15 dph when yolk reserves were largely depleted. Following exogenous feeding, expression of this gene may not decrease and larval growth is maintained. Further studies are needed to confirm the role of *myom2* in growth in European eel larvae.

In our study, most neural development-terms (*d2br*, *protocadherin-16*, *mt1r*, *adam22*, *pnx*, *otx2*) had low fold change expression in non-viable vs. viable larvae. Among them, *d2br* showed the lowest fold-change in non-viable vs. viable larvae (−9.4 fold) as well as to reduce motor behaviour in zebrafish larvae [102]. In addition, treating early zebrafish larvae with domperidone, a D2 receptor antagonist, increases larval activity [103]. The low fold changes of *d2br* in non-viable vs. viable larvae, but also the lack of *mtr1* that is essential for reducing locomotor behavior in zebrafish larvae [102,103,104], suggest that non-viable larvae differ in movement and active behavior from the viable larvae. For the other neural development-terms, studies have shown that *pnx* promotes neurogenesis in zebrafish [105] and *otx2* is essential for head speciation in pufferfish [106]. The low fold change of genes related to neural development in non-viable vs. viable larvae suggests that the non-viable larvae might have neural impairment, which is also indicated by the high fold change expression of *neurogulin-1* (6-fold) that is essential for peripheral nerve development and nerve repair in mice [107]. Little is known about the factors influencing early brain development during the yolk-sac stage in fish. To our knowledge, only the importance of exercise on neurogenic brain growth has been illustrated in larval zebrafish [108]. Further studies should investigate potential factors (e.g., inflammation) that could influence early brain development in European eels for improving eel larviculture.

Three genes (*lhx2*, *norrin*, *tmc1*) and one gene (*vertebrate ancient opsin*) related to sensory development showed low and moderate fold change in non-viable vs. viable larvae, respectively. Little is known about the functional role of *lhx2*, *norrin*, and *tmc1* in fish and thus further studies are needed to comprehend their role during early ontogeny. The physiological function of the *vertebrate ancient opsin* that has been described in several teleost fish [109,110,111,112] still remains to be elucidated but it might include irradiance detection tasks [112]. These four DEGs related to the sensory system were already expressed in 1 dph larvae, which is in agreement with the study of Sarropoulou et al. [113], who showed that many genes associated with the visual system were upregulated just after hatching in gilthead seabream. In European eels, the eyes are visible in 32 hpf embryos, start to pigment at 8 dph and become well-developed at 10 dph at 20 °C [6]. Unlike vision, the mechanosensory system is probably already functional at hatching since neuromasts were present on the head of 1 dph eel larvae between 18–23 °C in shortfinned eels [25]. In fish larvae, the development of sense organs will be essential for exogeneous feeding [114,115].

When considering that several differentially expressed genes related to myogenesis, neurogenesis, and sensory development had low fold change (lower than −4 fold) in non-viable vs. viable larvae, we can conclude that these processes are reduced in non-viable larvae. It appears that the non-viable larvae invested large amounts of their available energy in fighting against infection and maintaining homeostasis at the cost of normal development.

### 4.4. Digestive Function and Hyaluronan Metabolism

Numerous transcripts associated with digestive function, metabolism, and growth were highly abundant in 1 dph larvae, but were not differentially expressed in non-viable vs. viable larvae, suggesting that these biological processes are not impaired in non-viable larvae at 1 dph. Although the digestive system is still largely undifferentiated in new hatchings [6,27], expression of digestive enzymes is already detected just after hatching in European eels [32]. In our study, expression of lipid and protein digestion enzymes was higher than of carbohydrate digestion enzymes at 1 dph, indicating that larvae have a predisposition for proteins and lipids just after hatching. Literature about the nutritional predisposition of eel larvae shows discrepancy [27,31,32] and thus should be clarified by studying the digestive function during early ontogeny via transcriptomics to get a better overview. We also found that numerous genes involved in hyaluronan metabolism showed very high expression in 1 dph larvae. These findings are in accordance with the study of Okamura et al. [116] in which hyaluronan was detected soon after hatching in *A. japonica* larvae. Hyaluronan in the bodies of eel larvae is essential for growth and metamorphosis [116] and might regulate buoyancy due to its water-holding capacity [117]. When considering that expression of genes related to growth and metabolism did not differ between non-viable vs. viable larvae, we can conclude that these processes did not majorly contribute to the larval viability at this stage.

### 4.5. Activity: Movement and Stress

The temporal expression profiles observed here for *d2br*, which steadily increased during early ontogeny, agree with a previous study on zebrafish larvae [103]. Little is known about the role of D2br in eel larvae, but studies on zebrafish larvae suggest an important role of D2br in modulating the motor behavior [102,103]. In European eels, swimming activity increases from 8 dph onwards [6,118]. Furthermore, older larvae (13, 15, and 17 dph) swim actively by undulations of the caudal region and increase their attacks to food particles in the presence of various diets [118]. The upregulation of the *d2br* through early ontogeny is probably related to swimming activity that might be essential for active exogeneous feeding around 12–14 dph.

Like *d2br*, the temporal expression of *mtr1* steadily increased through early ontogeny. In vertebrates, melatonin is secreted primarily by the pineal gland during the dark period of the circadian cycle and is involved in many biological processes such as blood pressure regulation and circadian entrainment, as reviewed by [119]. Like *d2br*, melatonin regulates motor behavior in zebrafish larvae [102,103,104]. Melatonin possibly even influences the *d2br* transcript levels since high day/low night variation of *d2br* have been observed in adult eels [120]. The daily variations of the dopaminergic and melatonergic systems in eel larvae were beyond the scope of our study, but should be further investigated.

The temporal expression of *hspb1* also increased through early ontogeny in our study. Expression peaked at 15 dph, which corresponded to the start of exogeneous feeding in European eels [118]. In fish, *hspb1* is highly induced in response to stress as induced by temperature, pollution, and UV-B radiation, as reviewed by [121].

When considering the temporal expression of *d2br* and *mtr1* that significantly increase during early ontogeny and their role in modulating motor behavior in zebrafish fish larvae, we can assume that both genes reflect locomotion in European eel larvae. Since *hspb1* peaks at 15 dph when yolk-reserves are depleted, this gene might be induced in response to stress as induced by food deprivation. Heat-shock proteins are induced by food deprivation in other fish species [122,123]. The increase of these genes during early ontogeny might reflect the overall increase in activity at the start of active swimming (8 dph) and feed searching behavior (15 dph).

## 5. Conclusions

In European eel, larvae exhibit immune competency, which is in sharp contrast with the hypothesized immunocompromised period of Miest et al. [13]. Non-viable larvae initiated an immune response as they probably suffered from microbial infection. Non-viable larvae tried to maintain ionic and water homeostasis by strong osmoregulatory adaptations. Microbial control and salinity reduction might benefit eel larvae in terms of lower mortality and improved development by lowering the energetic costs of immune response and osmoregulation. The temporal expression patterns of *d2br*, *mtr1*, and *hspb1* in 1, 8, and 15 dph larvae reflect the increase in overall activity at the start of active swimming (8 dph) and feed searching behavior (15 dph).

## Figures and Tables

**Table 1:** Non-viable and viable larvae used for RNA-Seq analysis. Hatching time was expressed in hours post fertilization (hpf). Larvae viability was estimated by larvae longevity in days post-hatch (dph) to classify non-viable and viable larvae used for RNA-Seq.

Female ID	Hatch (hpf)	Longevity (dph)	Larvae Viability
847C	51	1	Non-viable
B43C	51	3	Non-viable
F8E1	72	1	Non-viable
044B	52	16	Viable
D785	60	22	Viable
AB79	59	7	Viable

**Table 2:** Primers used for each target gene with Abv: abbreviation; G: sequence obtained from the *A. anguilla* genome [60,61]; T°: annealing temperature and bp: base pair.

Target Genes	Abv.	Accession Number	Primer Sequences	T°	Product Size (bp)	Efficiency (%)
60s ribosomal protein l36	L36	G	FW: CCTGACCAAGCAGACCAAGT	62	160	91
			RV: TCTCTTTGCACGGATGTGAG			
Dopamine receptor-2B	D2br	DQ789977	FW: CACGCTACAGCTCCAAAAGAA	60	186	92
			RV: TGAAGGGGACATAGAAGGACAC			
Melatonin receptor	MT1R	Contig sequence	FW: CGACAAGAACTCCCTGTGCTA	62	175	92
			RV: CAGGATGAAGTGGAAGAAGACC			
M-protein	Myom2	Contig sequence	FW: GATGCAAAGATCACTCAGTCCA	62	160	106
			RV: CGTATTTGCCTTTGTCCTTCTC			
MHC-I	MHCI	Contig sequence	FW: CATGTGACCGGATTCTACCC	64	200	91
			RV: TGTTTCAGGCTCTTGTGCTGT			
Hspb1	Hspb1	Contig sequence	FW: GGGGCATATCCGAGATCAA	62	104	93
			RV: GACTCCATCCCTGGTCTTCAC			

**Table 3:** Genes associated with the immune response that are differentially expressed in non-viable larvae in comparison with viable larvae in European eel *Anguilla anguilla*. Both groups represent larvae samples taken 1 dph after which non-viable larvae survived less than 3 dph while viable larvae survived for at least a week up to 22 dph.

Description	Fold Change
Interleukin-17C	29.0
Complement decay-accelerating factor isoform X1	5.2
Pentraxin fusion protein-like	2.9
Nlrp 12 (NACHT, LRR and PYD domains-containing protein 12-like)	2.8
Leukocyte elastase inhibitor	2.6
Arginase-2, mitochondrial	2.3
Complement factor H-like	2.3
Complement component C7	−7.6
MHC1 (Class I histocompatibility antigen, F10 alpha chain-like)	−17.7

**Table 4:** Genes associated with osmoregulation that are differentially expressed in non-viable larvae in comparison with viable larvae in European eel *Anguilla anguilla*. Both groups represent larvae samples taken 1 dph, after which non-viable larvae survived less than 3 dph while viable larvae survived for at least a week up to 22 dph.

Description	Fold Change
Extracellular calcium-sensing receptor-like	39.4
Claudin-4	7.1
Sodium/myo-inositol cotransporter-like	3.9
Guanylin precursor	2.9
Claudin-10-like isoform X2	2.8
Claudin-16-like isoform X1	−7.9

**Table 5:** Genes associated with morphogenesis that are differentially expressed in non-viable larvae in comparison with viable larvae in European eel *Anguilla anguilla*. Both groups represent larvae samples taken 1 dph, after which non-viable larvae survived less than 3 dph while viable larvae survived for at least a week up to 22 dph.

Description	Fold Change
**Muscle development**	
Heat-shock protein beta-1 like	5.9
cGMP-dependent protein kinase 1-like	−4.0
Troponin I slow skeletal muscle-like	−4.8
Myom2 (M-protein, striated muscle-like isoform X1)	−10.5
**Neural development**	
Pro-neuregulin-1, membrane-bound isoform X1	6.7
Csmd3 (CUB and sushi domain-containing protein 3)	2.6
Homeobox protein OTX2	−2.3
Homeobox protein pnx-like isoform X1	−2.3
Adam22 (disintegrin and metalloproteinase domain-containing protein 22)	−2.5
MT1R (MT1 melatonin receptor)	−3.4
Protocadherin-16-like, partial	−5.6
D2br (dopamine D2B receptor)	−9.4
**Sensory system**	
Vertebrate ancient opsin-like	3.2
LIM/homeobox protein Lhx2	−2.2
Norrin-like	−2.5
Tmc1 (putative transmembrane channel-like protein 1)	−3.0
**Wnt signaling**	
Norrin-like	−2.5
Ptpro (Receptor-type tyrosine-protein phosphatase O, partial)	−4.3
CXXC-type zinc finger protein 4	−9.0
**Various functions**	
T-box transcription factor TBX1 isoform X4	2.3
Homeobox protein DLX-6	−2.4
Homeobox protein DLX-2	−2.4

## Data Availability

Raw RNA-Seq data (reads) have been submitted to NCBI’s SRA database with reference PRJNA735388 (http://www.ncbi.nlm.nih.gov/bioproject/735388; SAMN19580333-SAMN19580338; Temporary Submission ID: SUB9805749; Release date: 6 June 2021). Other raw data supporting the conclusions of this article will be made available by the authors, without undue reservation, to any qualified researcher.

## Supplementary Materials

The following are available online at https://www.mdpi.com/article/10.3390/ani11061710/s1, Table S1: Non-differentially expressed genes between non-viable vs. viable larvae, Table S2: Differentially expressed genes between non-viable vs. viable larvae, Table S3: Differentially expressed genes associated with the immune response, osmoregulation, muscle development, neural development, sensory development, Wnt signaling, and other various functions.

## References

[1] Fontaine M. (1936). Sur la maturation complète des organes génitaux de l’anguille male et l’emission spontanèe de ses produits sexuels. Comptes Rendus Acad. Sci..

[2] Fontaine M., Bertrand E., Lopez E., Callamand O. (1964). Sur la maturation des organes génitaux de l’anguille femelle (*Anguilla anguilla* L.) et l’émission spontanée des œufs en aquarium. Comptes Rendus Acad. Sci..

[3] Boëtius I., Boëtius J. (1980). Experimental maturation of female silver eels, *Anguilla anguilla*. Estimates of fecundity and energy reserves for migration and spawning. Dana.

[4] Bezdenezhnykh V.A., Prokhorchik G.A., Petrikov A.M., Petukov V.B., Plyuta M.V. (1983). Obtaining the larvae of European eel *Anguilla anguilla* L. (Pisces, *Anguillidae*) under experimental conditions. Dokl. Akad. Nauk SSSR.

[5] Mordenti O., Di Biase A., Bastone G., Sirri R., Zaccaroni A., Parmeggiani A. (2013). Controlled reproduction in the wild European eel (*Anguilla anguilla*); two populations compared. Aquac. Int..

[6] Sørensen S.R., Tomkiewicz J., Munk P., Butts I.A.E., Nielsen A., Lauesen P., Graver C. (2016). Ontogeny and growth of early life stages of captive-bred European eel. Aquaculture.

[7] Asturiano J.F., Sørensen S.R., Perez L., Lauesen P., Tomkiewicz J. (2016). First production of larvae using cryopreserved sperm: Effects of preservation temperature and cryopreservation on European eel sperm fertilization capacity. Reprod. Domest. Anim..

[8] Jéhannet P., Heinsbroek L.T.N., Palstra A.P. (2017). Ultrasonography to assist with timing of spawning in European eel. Theriogenology.

[9] Sørensen S.R., Skov P.V., Lauesen P., Tomkiewicz J., Bossier P., De Schryver P. (2014). Microbial interference and potential control in culture of European eel (*Anguilla anguilla*) embryos and larvae. Aquaculture.

[10] Da Silva F.F.G., Jacobsen C., Kjørsvik E., Støttrup J.G., Tomkiewics J. (2018). Oocyte and egg quality indicators in European eel: Lipid droplet coalescence and fatty acid composition. Aquaculture.

[11] Masuda Y., Imaizumi H., Oda K., Hashimoto H., Usuki H., Teruya K. (2012). Artificial completion of the Japanese eel, *Anguilla japonica*, life cycle: Challenge to mass production. Bull. Fish. Res. Agency.

[12] Okamura A., Horie N., Yamada Y., Mikawa N., Tsukamoto K. (2020). Obtaining high-quality larvae for mass production of glass eels: Can we refine our approach?. Aquaculture.

[13] Vadstein O., Bergh O., Gatesoupe F.G., Galindo-Villegas J., Mulero V., Picchietti S., Makridis P., Olsen Y., Dierckens K., Defoirdt T. (2013). Microbiology and immunology of fish larvae. Rev. Aquac..

[14] Vadstein O., Attramdal K.J.K., Bakke I., Olsen Y. (2018). K-selection as microbial community management strategy: A method for improved viability of larvae in aquaculture. Front. Microbiol..

[15] Miest J.J., Politis S.N., Adamek M., Tomkiewicz J., Butts I.A.E. (2019). Molecular ontogeny of larval immunity in European eel at increasing temperatures. Fish Shellfish Immun..

[16] Varsamos S., Nebel C., Charmantier G. (2005). Ontogeny of osmoregulation in postembryonic fish: A review. Comp. Biochem. Physiol. Part A Mol. Integr. Physiol..

[17] Sasai S., Kaneko T., Tsukamoto K. (1998). Extrabranchial chloride cells in early life stages of the Japanese eel, *Anguilla japonica*. Ichthyol. Res..

[18] Ahn H., Lee K.M., Inokuchi M., Watanabe S., Okamura A., Tsukamoto K., Kaneko T. (2015). Observations of initial water ingestion and ion absorption in the digestive tract of Japanese eel larvae. Fish Sci..

[19] Bagarino T. (1986). Yolk resorption, onset of feeding and survival potential of larvae of three tropical marine fish species reared in the hatchery. Mar. Biol..

[20] Lasker R. (1962). Efficiency and rate of yolk utilization by developing embryos and larvae of the Pacific sardine, *Sardinops caerulea* (Girard). J. Fish. Res. Board Can..

[21] Avila E.M., Juario J.V. (1987). Yolk and oil globule utilization and developmental morphology of the digestive tract epithelium in larval rabbitfish, *Siganus guttatus* (Bloch). Aquaculture.

[22] Xia J.H., Liu J.X., Zhou L., LI Z., Gui J.F. (2008). Apo-14 is required for digestive system organogenesis during fish embryogenesis and larval development. Int. J. Dev. Biol..

[23] Mazurais D., Darias M., Zambonino-Infante J.L., Cahu C.L. (2011). Transcriptomics for understanding marine fish larval development. Can. J. Zool..

[24] Yamauchi K., Nakamura M., Takahashi H., Takano K. (1976). Cultivation of larvae of Japanese eel. Nature.

[25] Lokman P.M., Young G. (2000). Induced spawning and early ontogeny of New Zealand freshwater eels (*Anguilla dieffenbachii* and *A. australis*). N. Z. J. Mar. Freshw. Res..

[26] Zambonino-Infante J.L., Gisbert E., Sarasquete S., Navarro I., Gutierrez J., Cahu C.L., Cyrino J.E.P., Bureau D., Kapoor B.D. (2008). Ontogeny and physiology of the digestive system of marine fish larvae. Feeding and Digestive Functions in Fishes.

[27] Kurokawa T., Suzuki T., Ohta H., Kagawa H., Tanaka H., Unuma T. (2002). Expression of pancreatic enzyme genes during the early larval stage of Japanese eel *Anguilla japonica*. Fish. Res..

[28] Mazurais D., Kjørsvik E., World P.A., Politis S.N., Cahu C., Tomkiewicz J., Zambonino-Infante J. Biochemical, histological and molecular study of digestive tract development in European eel larvae (*Anguilla anguilla*) prior to exogenous feeding. Proceedings of the Aquaculture Europe 13.

[29] Bouillart M., Tomkiewicz J., Lauesen P., De Kegel B., Adriaens D. (2015). Musculoskeletal anatomy and feeding performance of pre-feeding engyodontic larvae of the European eel (*Anguila anguilla*). J. Anat..

[30] Masuda Y., Shima Y., Tamaru O., Takahashi Y., Ohmura Y., Takashi I., Kamoshida M., Arimoto M., Yamano K., Yatabe T. (2019). Japanese eel jaw and vertebra ossification occurring respectively during the larval stage and metamorphosis. Fish. Sci..

[31] Politis S.N., Sørensen S.R., Mazurais D., Servili A., Zambonino-Infante J., Miest J.J., Clemmesen C.M., Tomkiewicz J., Butts I.A.E. (2018). Molecular ontogeny of first-feeding European eel larvae. Front. Physiol..

[32] Parmeggiani A., Zannoni A., Tubon I., Casalini A., Emmanuele P., Forni M., Mordenti O. (2020). Initial ontogeny of digestive enzymes in the early life stages of captive-bred European eels during fasting: A partial characterization. Res. Vet. Sci..

[33] Bishop R.E., Torres J.J. (1999). Leptocephalus energetics: Metabolism and excretion. J. Exp. Biol..

[34] Pfeiler E., Govoni J. (1993). Metabolic rates in early life history stages of Elopomorph fishes. Biol. Bull..

[35] Politis S.N., Butts I.A.E., Tomkiewicz J. (2014). Light impacts embryonic and early larval development of the European eel, *Anguilla anguilla*. J. Exp. Mar. Biol. Ecol..

[36] Politis S.N., Mazurais D., Servili A., Zambonino-Infante J.L., Miest J.J., Sorensen S.R., Tomkiewicz J., Butts I.A.E. (2017). Temperature effects on gene expression and morphological development of European eel, *Anguilla anguilla* larvae. PLoS ONE.

[37] Furuita H., Ohta H., Unuma T., Tanaka H., Kagawa H., Suzuki N., Yamamoto T. (2003). Biochemical composition of eggs in relation to egg quality in the Japanese eel, *Anguilla japonica*. Fish Physiol. Biochem..

[38] Furuita H., Unuma T., Nomura K., Tanaka H., Okuzawa K., Sugita T., Yamamoto T. (2006). Lipid and fatty acid composition of eggs producing larvae with high survival rate in the Japanese eel. J. Fish Biol..

[39] Kurokawa T., Okamoto T., Gen K., Uji S., Murashita K., Unuma T., Nomura K., Matsuraba H., Kim S.K., Ohta H. (2008). Influence of water temperature on morphological deformities in cultured larvae of Japanese eel, *Anguilla japonica*, at completion of yolk resorption. J. World Aquac. Soc..

[40] Okamura A., Yamada Y., Horie N., Utoh T., Mikawa N., Tanaka S., Tsukamoto K. (2007). Effects of water temperature on early development of Japanese eel *Anguilla japonica*. Fish. Sci..

[41] Okamura A., Yamada Y., Mikawa N., Horie N., Utoh T., Kaneko T., Tanaka S., Tsukamoto K. (2009). Growth and survival of eel leptocephali (*Anguilla japonica*) in low-salinity water. Aquaculture.

[42] Hsu H.Y., Chen S.H., Cha Y.R., Tsukamoto K., Lin C.Y., Han Y.S. (2015). *De Novo* Assembly of the Whole Transcriptome of the Wild Embryo, Preleptocephalus, Leptocephalus, and Glass Eel of *Anguilla japonica* and Deciphering the Digestive and Absorptive Capacities during Early Development. PLoS ONE.

[43] Izumi H., Gen K., Lokman P.M., Hagihara S., Horiuchi M., Tanaka T., Ijiri S., Adachi S. (2019). Maternal transcripts in good and poor quality eggs from Japanese eel, *Anguilla japonica*, their identification by large-scale quantitative analysis. Mol. Reprod. Dev..

[44] Chai Y., Tosaka R., Sago K., Hatanaka R., Ijiri S., Adachi S. (2010). The relationship between the developmental stage of oocytes in various seasons and the quality of the egg obtained by artificial maturation in the feminized Japanese eel *Anguilla Japonica*. Aquac. Sci..

[45] Mes D., Dirks R.P., Palstra A.P. (2016). Simulated migration under mimicked photothermal conditions enhances sexual maturation of farmed European eel (*Anguilla anguilla*). Aquaculture.

[46] Lokman P.M., Wylie M.J., Downes M., Di Biase A., Damsteegt E. (2015). Artificial induction of maturation in female eels, Anguilla australis: The benefits of androgen pre-treatment. Aquaculture.

[47] Thomson-Laing G., Damsteegt E.L., Nagata J., Ijiri S., Adachi S., Todo T., Hiramatsu N., Lokman P.M. (2019). Synergistic effects of estradiol and 11-ketotestosterone on vitellogenin physiology in the shortfinned eel (*Anguilla australis*). Biol. Reprod..

[48] Palstra A.P., Cohen E.G.H., Niemantsverdriet P.R.W., van Ginneken V.J.T., van den Thillart G.E.E.J.M. (2005). Artificial maturation and reproduction of European silver eel: Development of oocytes during final maturation. Aquaculture.

[49] Ohta H., Kagawa H., Tanaka H., Okuzawa K., Iinuma N., Hirose K. (1996). Artificial induction of maturation and fertilization in the Japanese eel, Anguilla Japonica. Aquaculture.

[50] Kahn I.A., Lopez E., Leloup-Hatey J. (1987). Induction of spermatogenesis and spermiation by a single injection of human chorionic gonadotropin in intact and hypophysectomised immature European eel (*Anguilla anguilla* L.). Gen. Comp. Endocrinol..

[51] Pérez L., Asturiano J.F., Tomás A., Zegrari S., Barrera R., Espinós F.J., Jover M. (2005). Induction of maturation and spermiation in the male European eel: Assessment of sperm quality throughout treatment. J. Fish Biol..

[52] Trapnell C., Pachter L., Salzberg S.L. (2009). TopHat: Discovering splice junctions with RNA-Seq. Bioinformatics.

[53] Li H., Handsaker B., Wysoker A., Fennell T., Ruan J., Homer N. (2009). 1000 Genome project data processing subgroup. The sequence alignment/map format and SAMtools. Bioinformatics.

[54] Anders S., Pyl P.T., Huber W. (2014). HTSeq-a Python framework to work with high-throughput sequencing data. Bioinformatics.

[55] Anders S., Huber W. (2010). Differential expression analysis for sequence count data. Genome Biol..

[56] Jéhannet P., Kruijt L., Damsteegt E.L., Swinkels W., Heinsbroek L.T.N., Lokman P.M., Palstra A.P. (2019). A mechanistic model for studying the initiation of anguillid vitellogenesis by comparing the European eel (*Anguilla anguilla*) and the shortfinned eel (*A. australis*). Gen. Comp. Endocrinol..

[57] Setiawan A.N., Lokman P.M. (2010). The use of reference gene selection programs to study the silvering transformation in a freshwater eel Anguilla australis: A cautionary tale. BMC Mol. Biol..

[58] Koressaar T., Remm M. (2007). Enhancements and modifications of primer design program Primer3. Bioinformatics.

[59] Untergasser A., Cutcutache I., Koressaar T., Ye J., Faircloth B.C., Remm M., Rozen S.G. (2012). Primer3–new capabilities and interfaces. Nucl. Acids Res..

[60] Henkel C.V., Burgerhout E., de Wijze D.L., Dirks R.P., Minegishi Y., Jansen H.J., Spaink H.P., Dufour S., Weltzien F.-A., Tsukamoto K. (2012). Primitive duplicate hox clusters in the European eel’s genome. PLoS ONE.

[61] Jansen H.J., Liem M., Jong-Raadsen S.A., Dufour S., Weltzien F.A., Swinkels W., Koelewijn A., Palstra A.P., Pelster B., Spaink H.P. (2017). Rapid de novo assembly of the European eel genome from nanopore sequencing reads. Sci. Rep..

[62] Bustin A.S., Benes V., Garson J.A., Hellemans J., Huggett J., Kubista M., Mueller R., Nolan T., Pfaffl M., Shipley G.L. (2009). The MIQE guidelines: Minimum information for publication of quantitative real-time PCR experiments. Clin. Chem..

[63] Livak K.J., Schmittgen T.D. (2001). Analysis of relative gene expression data using real-time quantitative PCR and the 2^−ΔΔCT^ method. Methods.

[64] Tanaka Y., Satoh K., Yamada H., Takebe T., Nikaido H., Shiozawa S. (2008). Assessment of the nutritional status of field-caught larval Pacific bluefin tuna by RNA/DNA ratio based on a starvation experiment of hatchery-reared fish. J. Exp. Mar. Biol. Ecol..

[65] Bobe J., Labbé C. (2010). Egg and sperm quality in fish. Gen. Comp. Endocrinol..

[66] Zhou Y., Liu Y., Luo Y., Zhong H., Huang T., Liang W., Xiao J., Wu W., Li L., Chen M. (2020). Large-scale profiling of the proteome and dual transcriptome in Nile tilapia (*Oreochromis niloticus*) challenged with low- and high-virulence strains of Streptococcus agalactiae. Fish Shellfish Immunol..

[67] Shen Y., Zhang J., Xu X., Fu J., Li J. (2012). Expression of complement component C7 and involvement in innate immune responses to bacteria in grass carp. Fish Shellfish Immunol..

[68] Wang S., Gao Y., Shu C., Xu T. (2015). Characterization and evolutionary analysis of duplicated C7 in miiuy croaker. Fish Shellfish Immunol..

[69] Guo B., Wu C., Lv Z., Liu C. (2016). Characterization and expression analysis of two terminal complement components: C7 and C9 from large yellow croaker, *Larimichthys crocea*. Fish Shellfish Immunol..

[70] Sun G., Li H., Wang Y., Zhang B., Zhang S. (2010). Zebrafish *complement factor H* and its related genes: Identification, evolution, and expression. Funct. Integr. Genom..

[71] Qi P., Wu B., Guo B., Zhanf C., Xu K. (2018). The complement factor H (CFH) and its related protein 2 (CFHR2) mediating immune response in large yellow croaker *Larimichthys crocea*. Dev. Comp. Immunol..

[72] Forlenza M., Fink I.R., Raes G., Wiegertjes G.F. (2011). Heterogeneity of macrophage activation in fish. Dev. Comp. Immunol..

[73] Chen L., Huang R., Zhu D., Wang Y., Mehjabin R., Li Y., Liao L., He L., Zhu Z., Wang Y. (2019). Cloning of six serpin genes and their responses to GCRV infection in grass carp (*Ctenopharyngodon idella*). Fish Shellfish Immunol..

[74] Zhu J., Fu Q., Ao Q., Tan Y., Luo Y., Jiang H., Li C., Gan X. (2017). Transcriptomic profiling analysis of tilapia (*Oreochromis niloticus*) following *Streptococcus agalactiae* challenge. Fish Shellfish Immun..

[75] Li M.F., Sun L. (2018). Characterization of a teleost membrane-associated protein that is involved in the regulation of complement activation and bacterial infection. Dev. Comp. Immunol..

[76] Koronega H., Kono T., Sakai M. (2010). Isolation of seven IL-17 family genes from the Japanese pufferfish *Takifugu rubripes*. Fish Shellfish Immun..

[77] Wang T., Martin S.A.M., Secombes C.J. (2010). Two interleukin-17C-like genes exist in rainbow trout *Oncorhynchus mykiss* that are differentially expressed and modulated. Dev. Comp. Immunol..

[78] Ding Y., Ao J., Chen X. (2017). Comparative study of interleukin-17C (IL-17C) and IL-17D in large yellow croaker *Larimichthys crocea* reveals their similar but differential functional activity. Dev. Comp. Immunol..

[79] Hulpke S., Tampé R. (2013). The MHC I loading complex: A multitasking machinery in adaptive immunity. Trends Biochem. Sci..

[80] Müller-Eberhard H.J. (1986). The membrane attack complex of complement. Annu. Rev. Immunol..

[81] Wang T., Zhang J. (2016). CsPTX1, a pentraxin of *Cynoglossus semilaevis*, is an innate immunity factor with antibacterial effects. Fish Shellfish Immunol..

[82] Barbé F., Douglas T., Saleh M. (2014). Advances in Nod-like receptors (NLR) biology. Cytokine Growth Factor Rev..

[83] Holland M.C.H., Lambris D. (2002). The complement system in teleosts. Fish Shellfish Immun..

[84] Cordero H., Brinchmann M.F., Cuesta A., Meseguer J., Esteban M.A. (2015). Skin mucus proteome map of European sea bass (*Dicentrarchus labrax*). Proteomics.

[85] Wiegertjes G.F., Wentzel A.S., Spaink H.P., Elks P.M., Fink I.R. (2016). Polarization of immune responses in fish: The ‘macrophages first’ point of view. Mol. Immunol..

[86] Fischer U., Dijkstra J.M., Kollner B., Kiryu I., Koppang E.O., Hordvik I., Sawamoto Y., Ototake M. (2005). The ontogeny of MHC class I expression in rainbow trout (*Oncorhynchus mykiss*). Fish Shellfish Immunol..

[87] Rodrigues P.N., Hermsen T.T., Van Maanen A., Taverne-Thiele A.A., Rombout J.H., Dixon B., Stet R.J. (1998). Expression of MhcCyca class I and class II molecules in the early life history of the common carp (*Cyprinus carpio* L.). Dev. Comp. Immunol..

[88] Lee K.M., Yamada Y., Okamura A., Tsukamoto K., Kaneko T. (2013). Hyposmoregulatory ability and ion- and water-regulatory mechanisms during the leptocephalus stages of Japanese eel *Anguilla japonica*. Fish. Sci..

[89] Okamura A., Yamada Y., Mikawa N., Horie N., Tsukamoto K. (2016). Effect of salinity on occurrence of notochord deformities in Japanese eel *Anguilla japonica* larvae. Aquac. Int..

[90] Politis S.N., Mazurais D., Servili A., Zambonino-Infante J.L., Miest J.J., Tomkiewicz J., Butts I.A.E. (2018). Salinity reduction benefits European eel larvae: Insights at the morphological and molecular level. PLoS ONE.

[91] Okamoto T., Kurokawa T., Gen K., Murashita K., Nomura K., Kim S.K., Matsubara H., Ohta H., Tanaka H. (2009). Influence of salinity on morphological deformities in cultured larvae of Japanese eel, *Anguilla japonica*, at completion of yolk resorption. Aquaculture.

[92] Fiol D.F., Kültz D. (2007). Osmotic stress sensing and signaling in fishes. FEBS J..

[93] Comrie M.M., Cutler C.P., Cramb G. (2001). Cloning and expression of guanylin from the European eel (*Anguilla anguilla*). Biochem. Biophys. Res. Commun..

[94] Yuge S., Inoue K., Hyodo S., Takei Y. (2003). A novel guanylin family (guanylin, uroguanylin, and renoguanylin) in eels: Possible osmoregulatory hormones in intestine and kidney. J. Biol. Chem..

[95] Kalujnaia S., Wilson G.D., Feilen A.L., Cramb G. (2009). Guanylin-like peptides, guanylate cyclase and osmoregulation in the European eel (*Anguilla anguilla*). Gen. Comp. Endocrinol..

[96] Marshall S., Breves J.P., Doohan E.M., Tipsmark C.K., Kelly S.P., Robertson G.N., Schulte P.M. (2018). *Claudin-10* isoform expression and cation selectivity change with salinity in salt-secreting epithelia of *Fundulus heteroclitus*. J. Exp. Biol..

[97] Schneider S. (2015). Inositol transport proteins. FEBS Lett..

[98] Tipsmark C.K., Luckenbach J.A., Madsen S.S., Kiilerich P., Borski R.J. (2008). Osmoregulation and expression of ion transport proteins and putative claudins in the fill of Southern Flounder (*Paralichthys lethostigma*). Comp. Biochem. Physiol. Part A.

[99] Agarkova I., Perriard J.C. (2005). The M-band: An elastic web that crosslinks thick filaments in the center of the sarcomere. Trends Cell. Biol..

[100] Farah C.S., Reinach F.C. (1995). The troponin complex and regulation of muscle contraction. FASEB J..

[101] Lincoln T.M., Dey N., Sellak H. (2001). Invited review: cGMP-dependent protein kinase signaling mechanisms in smooth muscle: From the regulation of tone to gene expression. J. Appl. Psychol..

[102] Souza B.R., Romano-Silva M.A., Tropepe V. (2011). Dopamine D2 receptor activity modulates Akt signaling and alters GABAergic neuron development and motor behavior in zebrafish larvae. J. Neurosci..

[103] Shontz E.C., Soulders C.L., Scmidt J.T., Martyniuk C.J. (2018). Domperidone upregulates dopamine receptor expression and stimulates locomotor activity in larval zebrafish (*Danio rerio*). Genes Brain Behav..

[104] Zhdanova I.V., Wang S.Y., Leclair O.U., Danilova N.P. (2001). Melatonin promotes sleep-like state in zebrafish. Brain Res..

[105] Bae Y.K., Shimizu T., Yabe T., Kim C.H., Hirata T., Nojima H., Muraoka O., Hirano T., Hibi M. (2003). A homeobox gene, *pnx*, is involved in the formation of posterior neurons in zebrafish. Development.

[106] Kimura-Yoshida C., Kitajima K., Oda-Ishii I., Tian E., Suzuki M., Yamamoto M., Suzuki T., Kobayashi M., Aizawam S., Matsuo I. (2004). Characterization of the pufferfish *Otx2* cis-regulators reveals evolutionarily conserved genetic mechanisms for vertebrate head specification. Development.

[107] Fricker F.R., Bennett D.L. (2011). The role of neuregulin-1 in the response to nerve injury. Future Neurol..

[108] Hall Z.J., Tropepe V. (2018). Movement maintains forebrain neurogenesis via peripheral neural feedback in larval zebrafish. Elife.

[109] Soni B.G., Foster R.G. (1997). A novel and ancient vertebrate opsin. FEBS Lett..

[110] Minamoto T., Shimizu I. (2002). A novel isoform of vertebrate ancient opsin in a smelt fish, *Plecoglossus altivelis*. Biochem. Biophys. Res. Commun..

[111] Kojima D., Mano H., Fukada Y. (2000). Vertebrate ancient-long opsin: A green sensitive photoreceptive molecule present in zebrafish deep brain and retinal horizontal cells. J. Neurosci..

[112] Philip A.E., Garcia-Fernandez J.M., Soni B.G., Lucas R.J., Foster B.R.G. (2000). Vertebrate ancient (VA) opsin and extraretinal photoreception in the Atlantic salmon (*Salmo salar*). J. Exp. Biol..

[113] Sarropoulou E., Kotoulas G., Power D., Geisler R. (2005). Gene expression profiling of gilthead sea bream during early development and detection of stress-related genes by the application of cDNA microarray technology. Physiol. Genom..

[114] Blaxter J.H.S. (1968). Light intensity, vision and feeding in young plaice. J. Exp. Mar. Biol. Ecol..

[115] Yahaya S., Lim L.H., Shaleh S.R.M., Mukai Y., Anraku K., Kawamura G. (2011). Ontogenetic eye development and related behavioural changes in larvae and juveniles of barramundi *Lates calcarifer* (Bloch). Mar. Freshw. Behav. Physiol..

[116] Okamura A., Sakamoto Y., Yamada Y., Tsukamoto K. (2018). Accumulation of hyaluronan in reared Japanese eel Anguilla japonica during early ontogeny. Aquaculture.

[117] Tsukamoto K., Yamada Y., Okamura A., Kaneko T., Tanaka H., Miller M.J., Horie N., Mikawa N., Utoh T., Tanaka S. (2009). Positive buoyancy in eel leptocephali: An adaptation for life in the ocean surface layer. Mar. Biol..

[118] Butts I.A.E., Sørensen S.R., Politis S.N., Tomkiewicz J. (2016). First-feeding by European eel larvae: A step towards closing the life cycle in captivity. Aquaculture.

[119] Li D.Y., Smith D.G., Hardeland R., Yang M.Y., Xu H.L., Zhang L., Yin H.D., Zhu Q. (2013). Melatonin receptor genes in vertebrates. Int. J. Mol. Sci..

[120] Byun J.H., Hyeon J.Y., Kim E.S., Kim S.K., Hur S.P., Kim S.J., Takemura A. (2020). Daily variation of D2 dopamine receptor transcription in the brain of the Japanese eel Anguilla japonica and its regulation with dopamine and melatonin. Comp. Biochem. Physiol. Part A Mol. Integr. Physiol..

[121] Mohanty B.P., Mahanty A., Mitra T., Parija S.C., Mohanty S., Asea A., Kaur P. (2018). Heat shock proteins in stress in teleosts. Regulation of Heat Shock Protein Responses.

[122] Yengkokpam S., Pal A.K., Sahu N.P., Jain K.K., Dalvi R., Misra S., Debnath D. (2008). Metabolic modulation in *Labeo rohita* fingerlings during starvation: *Hsp70* expression and oxygen consumption. Aquaculture.

[123] Dar S.A., Srivastava P.P., Varghese T., Nazir M.I., Gupta S., Krishna G. (2019). Temporal changes in superoxide dismutase, catalase, and heat shock protein 70 gene expression, cortisol and antioxidant enzymes activity of *Labeo rohita* fingerlings subjected to starvation and refeeding. Gene.

